# Insights into Finishing Defects in Abrasive Flow Machining of Turbine Blade Film Cooling Holes

**DOI:** 10.3390/mi17070847

**Published:** 2026-07-16

**Authors:** Jieguang Huang, Haoyu Zhong, Zhijun Wang, Tingting Xu, Lifei Wang

**Affiliations:** 1 School of Modern Post, Xi’an University of Posts & Telecommunications, Xi’an 710100, China; 2 School of Materials Science and Engineering, Northwestern Polytechnical University, Xi’an 710072, China; 3 School of Chemistry and Chemical Engineering, Northwestern Polytechnical University, Xi’an 710129, China; 4 Science and Technology on Advanced High Temperature Structural Materials Laboratory, AECC Beijing Institute of Aeronautical Materials, Beijing 100095, China

**Keywords:** abrasive flow machining, film cooling holes, finishing defects, superalloy, fluid dynamics, surface quality, material removal uniformity, cavitation

## Abstract

Abrasive flow machining (AFM) is an effective finishing process for complex internal surfaces, particularly cavities, intersecting holes, and micro-channels that are difficult to access using conventional tools. However, when low-viscosity abrasive media is used (here defined, relative to conventional putty-like viscoelastic AFM carriers (with apparent viscosities of 10^3^–10^5^ mPa·s), as a water-based slurry with an apparent viscosity below 300 mPa·s over the operating shear-rate range), unfavorable flow conditions during the initial polishing stage can induce local over-polishing, erosion depressions, stepped patterns, and cavitation pits, resulting in non-uniform surface quality. The relationship between these flow behaviors and polishing defects remains insufficiently understood. To address this issue, this study investigates the AFM process applied to turbine blade film cooling holes through combined experimental and numerical approaches. The observed defects include erosion depressions, stepped surface patterns, and cavitation pits. The effects of abrasive injection pressure, flow velocity, hole geometry, abrasive viscosity, and particle size on defect formation are systematically examined. The results show that the initial abrasive filling level strongly affects defect distribution by altering the evolution of shear fields and void regions within the hole. Experimentally, at high Reynolds numbers (*Re* > 2 × 10^4^), intensified local shear and cavitation promote defect formation, while a moderate inclination angle (45–60°) and a higher aspect ratio (>8) are favorable for polishing uniformity. Complementary numerical simulations further indicate that smaller abrasive particles (<5 μm) and a moderate abrasive viscosity (~60 mPa·s) are predicted to improve polishing uniformity. This study clarifies the fluid-dynamic origin of polishing defects in film cooling holes and provides process guidance for suppressing local over-polishing, cavitation, and uneven material removal.

## 1. Introduction

Since its development in the 1950s to meet the demanding cooling requirements of aircraft engine turbine blades, the film cooling technique has remained a critical technology in turbine blade design. By allowing a thin film of cooler air to flow over the blade surface, film cooling holes offer effective thermal protection, thereby significantly extending blade service life and reducing operating temperatures [1,2,3]. Modern aero-engine turbine blades now incorporate hundreds to thousands of film cooling holes with spatially graded distributions [4]. Nevertheless, a key challenge lies in the fact that laser drilling or electrical discharge machining processes often leave rough surfaces, recast layers, microcracks, and other defects detrimental to cooling performance and fatigue life [5,6,7]. Additionally, the sharp edges at both the inlet and outlet of the film cooling hole tend to induce stress concentrations, which can in turn promote the initiation of microcracks during service [8]. To improve the cooling effectiveness and structural strength, film cooling holes typically require post-processing to improve surface quality and to round sharp edges in order to mitigate peak stress concentration.

AFM is a non-conventional finishing process that uses a viscoelastic abrasive medium forced through internal passages of a workpiece under controlled pressure and flow conditions. The abrasive particles within the media remove material by grinding rather than shearing, enabling uniform polishing, deburring, and edge-rounding even in complex internal geometries inaccessible to conventional tools [9]. AFM’s ability to polish complex internal channels and holes makes it ideal for finishing film cooling holes with intricate shapes such as fan-shaped or shaped cooling holes designed to optimize cooling air diffusion [10]. The abrasive media flows through the holes, deburring and eliminating surface irregularities while preserving the original hole geometry, thereby enhancing cooling efficiency and component reliability [11,12].

Key parameters influencing AFM performance include process conditions such as polishing duration, flow rate, pressure, and tooling design that ensures uniform media distribution within the workpiece channels; the rheological properties of the media (e.g., viscosity and elasticity); and the size and concentration of abrasive particles [13]. Recent studies have systematically optimized AFM parameters to enhance finishing quality.

In terms of system process parameters, Maneepen et al. [14] demonstrated that increasing polishing cycles, extrusion force, and media temperature significantly improves surface finish on aluminum alloy internal channels, achieving sub-micron roughness levels. Cai et al. [15] proposed an adaptive pressure difference control system that maintains an optimal pressure differential (~0.28 MPa) between inlet and outlet, reducing surface roughness non-uniformity by 59.1% and eliminating surface scratches in cylindrical holes. They confirmed that excessive differential pressure compromises axial shear forces, thus reducing machining efficiency. Despite this, adaptive pressure control remains crucial for uniform polishing of the internal surfaces in cooling holes with high aspect ratios. Ye et al. [16] reported a novel shear thickening abrasive flow polishing method for medical titanium alloy micro-hole inner walls, established its dynamic and material removal models, and optimized key parameters (flow behavior index and velocity) to achieve high-efficiency, low-deformation polishing. Similarly, Tang et al. [17] revealed the mechanism of wall effects (including over-polishing at inlets) in abrasive flow machining, demonstrating that increasing hole diameter (>15 mm) or decreasing inlet velocity (<20 m/s) improves pressure uniformity, while lower initial roughness (*Ra* = 0.296 μm) reduces wall effects and higher roughness (*Ra* = 4.273 μm) enhances material removal rate.

In terms of abrasive media properties, through studying the effects of AFM on residual stresses in internal channels of 15-5PH stainless steel, Han et al. [18] found that AFM can induce compressive residual stress on the inner surface, with the type and size of abrasive grains playing a critical role. Duval-Chaneac et al. [19] examined the effects of different AFM media parameters on surface roughness and analyzed the wear mechanisms between abrasive grains and the SLM surface. The results showed that AFM media parameters significantly affect surface roughness evolution. Higher abrasive concentration and media viscosity lead to lower surface roughness values. Hashmi et al. [20] applied Taguchi design of experiments to optimize media viscosity and finishing time on FDM-printed hollow truncated cones, achieving a 94.26% improvement in surface roughness (from 20.93 μm to 1.20 μm). Yang et al. [21] developed a material removal model that combines single-sized and multi-sized abrasives. The comparative experiments on GH4169 alloy clearly demonstrate the advantages of multi-sized abrasives in achieving better surface roughness, removal depth, morphology, and uniformity.

To address limitations such as low material removal rates and uneven finishing, hybrid AFM techniques have been developed. Bhardwaj et al. [22] introduced a new hybrid process called thermal additive centrifugal force-assisted abrasive flow machining (TACAFM). They investigated the effects of process parameters on surface finish and material removal using a novel spline-shaped electrode with a curved blade. They suggest that the TACAFM process with the proposed electrode enhances material removal and surface finish compared to conventional AFM. Simulation and experimental results validate the effectiveness of the new electrode in improving the machining process. Choopani et al. [23] introduced ultrasonic-assisted rotational magnetorheological abrasive flow finishing (UA-RMRAFF), combining ultrasonic vibrations with magnetorheological fluid flow, achieving uniform nano-scale finishes (~25.5 nm) on aluminum alloy tubes. Dixit et al. [24] combined ultrasonic and magnetic field assistance to polish 3D printed polymer parts, achieving up to 54.42% surface roughness reduction. These hybrid techniques enhance abrasive particle interaction and finishing efficiency, broadening AFM’s applicability.

Beyond the aforementioned technological innovations, computational modeling of abrasive media flow and material removal mechanisms enables process optimization [25]. For instance, Dash and Maity [26] reported a detailed investigation of the AFM process using CFD simulations for both 2D and 3D models. The authors successfully developed a 2D model to analyze flow parameters, force calculations, and material removal predictions, validating the results with experimental data. The extension to a 3D model for a swaging die further demonstrates the applicability of CFD in understanding the effects of media viscosity on machining performance. Zhang et al. [27] investigated the material removal mechanisms in AFM applied to micro-channels with rough surfaces. They developed a physical and numerical model to describe how abrasives interact with tiny surface peaks. The effectiveness of the process was closely related to the rheological properties of the media. Peng et al. [28] developed a modified Preston equation based on concentrated suspensions theory, revealing that particle phase normal stress drives material removal uniformity in AFM of SLM internal channels, and demonstrated that increasing abrasive media viscosity enhances finishing quality despite geometric non-uniformities. These models aid in tooling design and parameter selection to maximize finishing uniformity.

Although considerable effort has been devoted to optimizing AFM, previous studies have consistently reported persistent non-uniform material removal in miniature film cooling holes with complex geometries, particularly when low-viscosity abrasive media are employed. For example, Tang et al. [17] showed that wall effects promote localized over-polishing near hole inlets, Cai et al. [15] demonstrated that uncontrolled pressure differentials reduce surface-finish uniformity, and Peng et al. [28] attributed non-uniform material removal in AFM of SLM-fabricated internal channels to particle-phase normal stress. Despite these advances, the fluid-mechanical mechanisms responsible for defect formation remain insufficiently understood, particularly the relationship between transient flow evolution and the spatial distribution of individual surface defects. The present study focuses on the formation mechanisms of hole-wall surface defects during AFM. The transient abrasive-filling process, including flow-front propagation, air entrapment, and void evolution, is first resolved using a VOF-based numerical model. The evolution of the near-wall shear field and cavitation characteristics is then correlated with the location and morphology of the three experimentally observed hole-wall surface defect types, namely erosion depressions, stepped patterns, and cavitation pits. Based on these mechanistic insights, process windows are established for injection pressure (Reynolds number), hole inclination angle, aspect ratio, particle size, and abrasive viscosity to suppress defect formation and improve material-removal uniformity.

## 2. Experimental Apparatus and Methods

**Figure 1:**
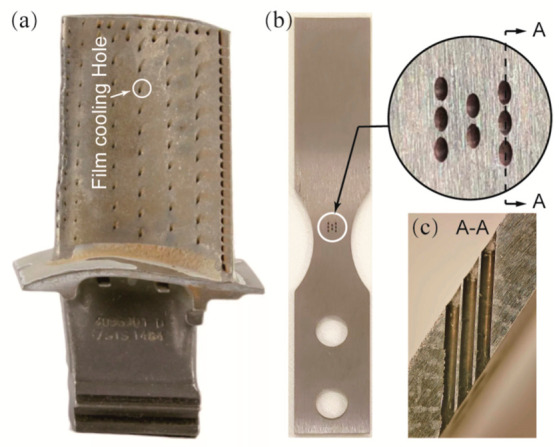
Film cooling hole samples: (**a**) illustration of a turbine blade with film cooling holes, (**b**) test workpiece, (**c**) cross-section of the film cooling hole test workpiece.

In this study, test workpieces were fabricated based on the geometric characteristics of film cooling holes in turbine blades (Figure 1a) to investigate the abrasive flow machining (AFM) finishing process. All workpieces were made from the same nickel-based superalloy as the actual turbine blades, and the holes were fabricated using the same femtosecond laser drilling technique employed in blade production. Eight film cooling holes, each with a diameter of 0.4 mm, were prepared. A cross-sectional view of a hole oriented at 60° to the surface normal is shown in Figure 1c.

To improve reproducibility, the geometric parameters of the film cooling holes prepared on each test workpiece are summarized in Table 1.

**Figure 2:**
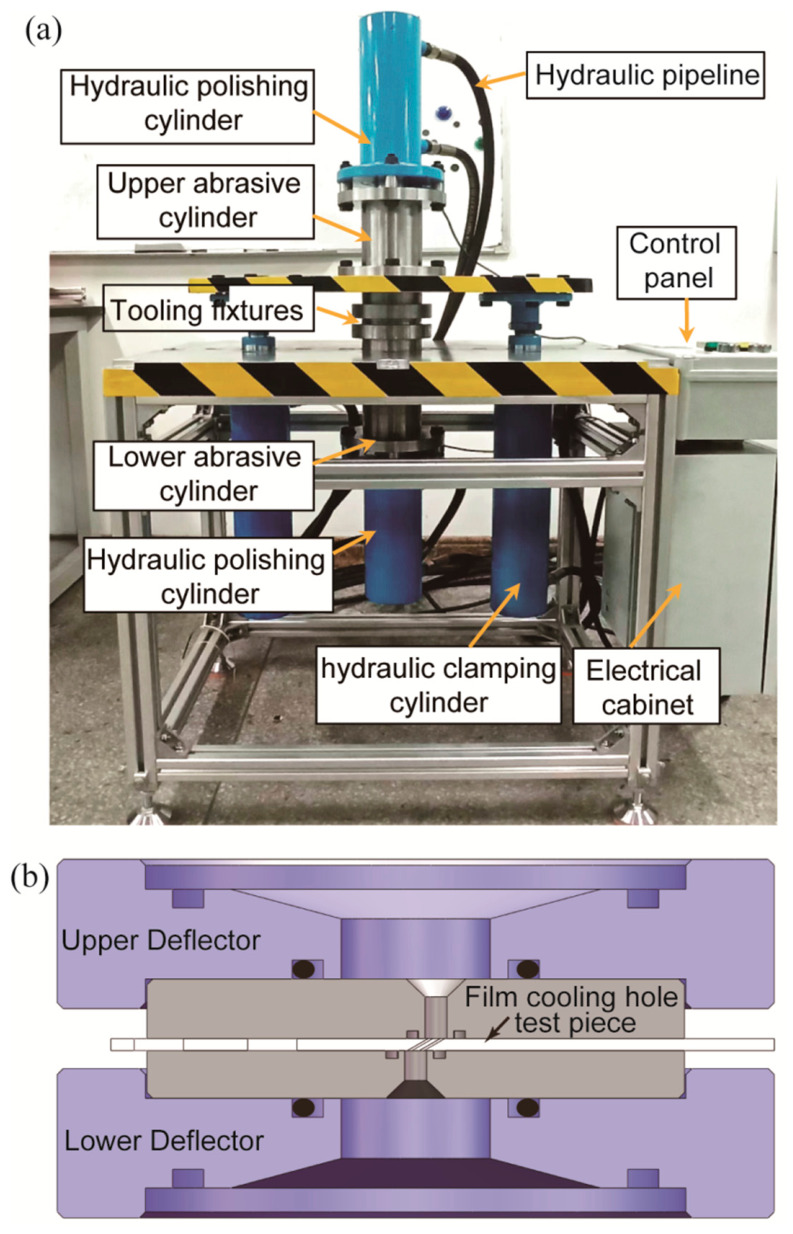
(**a**) Experimental setup. (**b**) Fixture of the film cooling hole test piece.

To facilitate abrasive flow machining of the film-cooling-hole workpiece shown in Figure 1b, a custom polishing test apparatus was designed and developed, as shown in Figure 2a. The system comprises several key subsystems, including a hydraulic power unit, a hydraulic clamping device, a reciprocating abrasive cylinder assembly, and specialized tooling fixtures. The test platform features a compact, bidirectional reciprocating flow configuration, with two symmetrically arranged vertical abrasive cylinders, enabling the use of both Bingham-type abrasive media and low-viscosity water-based slurries. Each reciprocating abrasive cylinder is powered by an individual hydraulic cylinder. The hydraulic cylinder is connected to an independent hydraulic circuit, including with a solenoid directional valve, a flow control valve, and a pressure regulating valve. Through the coordinated reciprocating motion of the upper and lower abrasive cylinders, the abrasive media is driven to flow back and forth through the film-cooling-hole specimen secured in the fixture, thereby achieving repeated polishing of the film cooling holes. To precisely control the polishing process parameters and evaluate the finishing performance of the abrasive media, an independent hydraulic control system and a real-time monitoring system were developed for the test platform. The hydraulic clamping device is driven by two synchronized hydraulic cylinders, which are connected in parallel to a single hydraulic circuit equipped with a solenoid directional valve, a flow control valve, and a pressure regulating valve.

In the experimental setup, key process parameters such as abrasive injection pressure and polishing speed can be precisely controlled via the pressure and flow control valves in each hydraulic circuit. Moreover, the hydraulic system supports both manual and automatic polishing modes. In manual mode, the synchronized movement of the abrasive cylinders can be independently controlled via mechanical push-buttons for stepwise operation. In automatic mode, limit switches monitor the stroke of both cylinders and trigger automatic directional switching, while a timer is used to control the polishing duration.

As shown in Figure 2b, the film-cooling-hole specimen is clamped within the fixture, which is assembled from upper and lower flow-directing plates and a set of clamps. Prior to polishing, the abrasive media is loaded into the lower abrasive cylinder, of which the maximum volume is 150 mL. The lower flow-directing plate of the fixture is aligned with the lower abrasive cylinder, and the hydraulic system is activated to extend the clamping-cylinder rods, driving the upper abrasive cylinder downward to secure the fixture. Finally, the polishing program is initiated: both upper and lower abrasive cylinders alternately drive the media back and forth, forcing it to flow from the lower cylinder, through the film cooling holes in the specimen, into the upper cylinder, and then back again, until the polishing cycle is complete.

**Figure 3:**
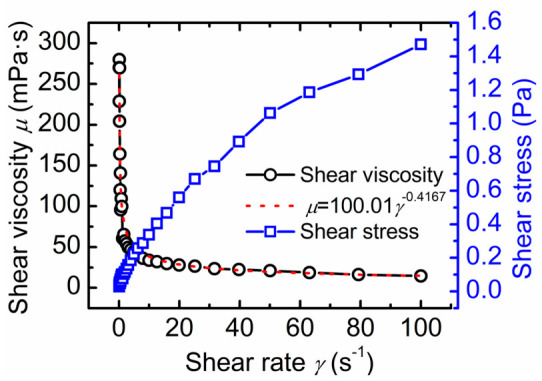
Viscosity and shear stress of the abrasive media versus shear rate.

The abrasive employed in these experiments is a self-developed formulation, whose physical properties are listed in Table 2. Its viscosity and shear stress as functions of shear rate are plotted in Figure 3. The shear-viscosity relationship is given by *μ* = 100.01*γ*^−0.4167^ (where *γ* denotes the shear rate, *γ* = *dV*_0_/*dr*), indicating that the media behaves as a shear-thinning non-Newtonian fluid. The apparent viscosity of the water-based SiC slurry varies from 20 mPa·s to 300 mPa·s at shear rates of 1–100 s^−1^, falling within the low-viscosity AFM regime. It should be noted that, throughout this paper, the term “low-viscosity” is defined relative to conventional viscoelastic AFM media (typically silicone- or polymer-based putties with an apparent viscosity of 10^3^–10^5^ mPa·s).

## 3. Theoretical Models

**Figure 4:**
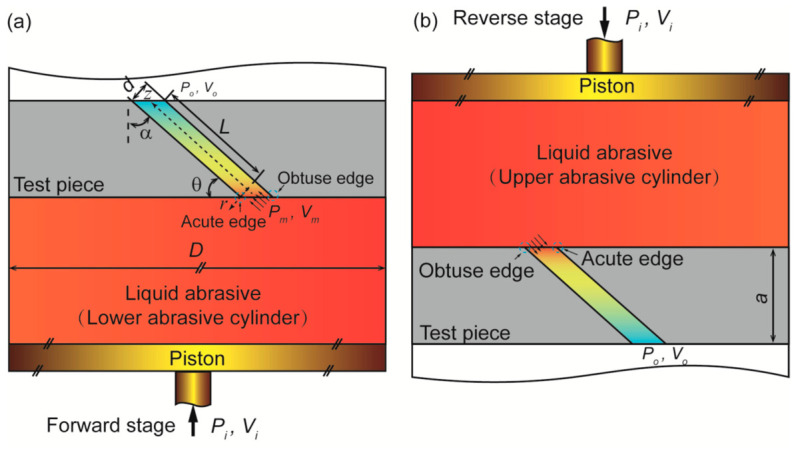
Schematic diagram of a film-cooling-hole polishing cycle: (**a**) forward stage, (**b**) reverse stage.

During the film-cooling-hole polishing process, when the abrasive flow has reached steady state, assume that the abrasive fully fills the flow passages within both the fixture and the workpiece film cooling holes, as shown in Figure 4. Let the abrasive cylinder diameter be *D*, the film-cooling-hole diameter be *d*, the specimen thickness be *a*, the piston velocity be *V_i_*, the cylinder pressure be *P_i_*, and the average velocity and pressure at the film-cooling-hole outlet be *V*_0_ and *P_o_*, respectively. Then, by the continuity equation, we have

(1) $$D^{2}V_{i}=d^{2}V_{0}$$

namely

(2) $$V_{0}={\left(\frac{D}{d}\right)}^{2}V_{i}$$

In film cooling holes abrasive flow finishing, the ratio of the abrasive cylinder piston diameter to the film cooling hole diameter (referred to as the cylinder-to-hole diameter ratio *k = D*/*d*) is commonly introduced as a measure of the polishing capability of the system. Accordingly, Equation (2) can be expressed as:

(3) $$V_{0}=k^{2}V_{i}$$

The hydraulic polishing cylinder in the experimental setup shown in Figure 2a has a diameter of 63 mm. The test workpiece contains eight film cooling holes, each with a diameter of ~0.4 mm. The equivalent diameter is 1.13 mm, resulting in a cylinder-to-hole diameter ratio of *k* = 55.8. In the experiment, the piston velocity *V_i_* was approximately set as 0.033 m/s, corresponding to an average outflow velocity of *V_o_* = 102.8 m/s.

When the abrasive flow reaches a stable state, it is assumed to be fully developed steady laminar flow (namely $\partial u_{z}/\partial z=0$). Neglecting the temperature variation of the abrasive media during the polishing process, the flow within the cylindrical film cooling hole is governed by the Navier-Stokes equation:

(4) $$\frac{\partial \left(\rho u_{z}\right)}{\partial t}+\frac{\partial P}{\partial z}-\mu \left[\frac{1}{r}\frac{\partial }{\partial r}\left(r\frac{\partial u_{z}}{\partial r}\right)+\frac{\partial^{2}u_{z}}{r\partial \theta^{2}}+\frac{\partial^{2}u_{z}}{\partial z^{2}}\right]-\rho g=0$$

and the continuity equation:

(5) $$\frac{\partial \rho }{\partial t}+\frac{\partial \rho u_{r}}{\partial r}+\frac{1}{r}\rho u_{r}+\frac{1}{r}\frac{\partial \rho u_{\theta }}{\partial \theta }+\frac{\partial \rho u_{z}}{\partial z}=0$$

Then, we made the following assumptions based on the real physical processes: (1) the abrasive media is incompressible (namely ∂ρ/∂t=0); and (2) the influence of gravity on fluid dynamics is small enough to be negligible (*ρ*·*g* = 0). Since the film-cooling-hole outlet is defined as an outflow boundary, we have *P_o_* = 0. Based on the above assumptions, Equation (5) can be simplified as

(6) $$\frac{\partial }{\partial r}(ru_{r})=0$$

Accordingly, there is $ru_{r}=C$ (*C* is constant). At the film-cooling-hole wall, the boundary condition is ${\left.u_{r}\right|}_{r=r_{1}}=0$. Accordingly, C=0 and $u_{r}\equiv 0$, and Equation (4) can be simplified as

(7) $$\frac{\partial P}{\partial z}+\mu \frac{1}{r}\frac{\partial }{\partial r}r\left(\frac{\partial u_{z}}{\partial r}\right)=0$$

Integrating the variable *r* in Equation (7) and applying the boundary condition $u_{z}=0\left|r=r_{1}\right.,u_{z}<\infty \left|r=0\right.$, we obtain

(8) $$u_{z}=\frac{P_{m}-P_{o}}{4L\mu }\left({\frac{d}{4}}^{2}-r^{2}\right)$$

Thus, the abrasive flow rate *Q* within the film cooling hole of diameter *d* can be expressed as:

(9) $$Q=\int_{0}^{\frac{d}{2}}2\pi ru_{z}dr=\frac{\pi d^{4}}{128L\mu }P_{m}$$

Based on geometric relationships, the effective length *L* of the film cooling hole in Equation (9) can be expressed as

(10) $$L=\frac{a-d\cos\theta }{\sin\theta }$$

Substituting Equation (10) into Equation (9), and applying the Bernoulli equation, we have

(11) $$V_{0}=V_{m}=\frac{d^{2}\sin\theta }{32\mu \left(a-d\cos\theta \right)}P_{m}$$

As the abrasive media flows from the abrasive cylinder into the film cooling hole, the reduction in flow cross-sectional area at the inlet results in the formation of a contraction neck and the onset of a vena contracta effect. Once the abrasive flow enters the internal channel of the film cooling hole, it gradually stabilizes. During this transition, a vortex region tends to form near the acute edge at the inlet. The interaction between the high velocity in the contraction region and the adverse pressure gradient in the downstream expansion zone induces turbulence and mechanical energy loss. The corresponding pressure loss can be expressed as

(12) $$\Delta P=P_{i}-P_{m}=K_{s}\frac{\rho {V_{m}}^{2}}{2}$$

where *K_s_* is pressure loss coefficient.

(13) $$K_{s}=\frac{1}{2}\left(1-{\left(\frac{C_{c}A}{A_{m}}\right)}^{2}\right)\cdot \delta$$

where *C_c_* is contraction coefficient, *C_c_* ≈ 0.62~0.64 (related to the Reynolds number *Re*), *A* denotes the cross-sectional area of the film cooling hole, while *A_m_* represents the inlet area of the inclined film cooling hole, given by *A_m_ = A*/Cos*θ*. Furthermore, under real operating conditions, the abrasive media flowing through the film cooling hole experiences pressure losses due to factors such as turbulence, vortex formation, shear layer detachment, and energy dissipation from abrasive particle collisions. To account for these effects, an additional pressure correction factor *δ* is introduced into the pressure loss coefficient formula. The value of *δ* can be determined through calibration based on experimental data from the system. Substituting Equation (13) into Equation (12) yields:

(14) $$P_{m}=P_{i}-\frac{1}{4}\delta \left(1-{C_{c}}^{2}\cos\theta^{2}\right)\rho {V_{0}}^{2}$$

Combining Equations (11) and (14), we have

(15) $$P_{i}=\frac{32\mu \left(a-d\cos\theta \right)}{d^{2}\sin\theta }V_{0}+\frac{1}{4}\delta \left(1-{C_{c}}^{2}\cos\theta^{2}\right)\rho {V_{0}}^{2}$$

Equation (15) implies the mapping between the injection pressure of the abrasive cylinder and the average fluid velocity of the abrasive flow in film cooling hole. Based on this model, the polishing velocity was regulated by adjusting the system’s hydraulic pressure, which should remain above the theoretical threshold *P_i_* to ensure effective media flow.

## 4. Numerical Models

During the abrasive flow machining process, the hydraulic system drives the abrasive media from the cylinder into the film cooling hole along the fixture channel. As the flow transitions from the abrasive cylinder to the film cooling hole, the cross-sectional area decreases sharply, causing the abrasive to accelerate and flow through the hole at a relatively high velocity. Thus, the inner wall of the film cooling hole is polished by the high-speed wall shear flow. Since the abrasive flow occurs within a closed channel formed by the fixture and the workpiece, direct observation of the flow field during polishing is not possible. Moreover, under current experimental conditions, capturing the flow dynamics using conventional instrumentation remains highly challenging. Non-invasive, high-resolution techniques, such as synchrotron X-ray imaging and densitometry, have recently enabled simultaneous measurements of velocity and void fraction in optically opaque, sub-millimeter multiphase flows [29]. These techniques offer a promising means of directly validating the bubble and particle trajectories predicted in Section 5. However, access to the required synchrotron facilities was not available in the present study. Therefore, numerical simulation is employed in this study to investigate the fluid dynamic behavior of the abrasive flow during the polishing process. A three-dimensional numerical model of film cooling hole abrasive flow machining was developed to analyze the flow field and its influence on polishing quality under various process conditions. The following assumptions are adopted in the model: (1) the abrasive media, being an aqueous solution with extremely low compressibility, is treated as an incompressible fluid; and (2) since the simulation focuses only on the transient flow development prior to stabilization, with a duration of less than 1 s, heat generation and the resulting temperature rise are assumed to be negligible. Therefore, an isothermal condition is adopted throughout the analysis.

Here, it should be noted that the analytical derivation assumes the abrasive media to be a steady, incompressible single-phase fluid for establishing the pressure-velocity relationship. This assumption applies only to the fully developed polishing stage, whereas the initial air-entrainment and void-evolution processes are resolved separately in the numerical model by the VOF method.

### 4.1. Governing Equations

The abrasive flow obeys the Navier–Stokes equation and the continuity equation. Based on the above assumptions, the Navier–Stokes equation and continuity equation can be expressed as follows:

(16) $$\frac{\partial U}{\partial t}+\left(U\cdot \nabla \right)U=-\frac{1}{\rho }\nabla P+\nabla^{2}\cdot \tau +f$$

(17) ∇U=0

where **U**, **P**, **τ** are the velocity vector, pressure and shear stress tensor, respectively. **f** represents the external force vector acting on the fluid, including gravity and surface tension.

Given that the Reynolds number *Re* of the abrasive flow inside the film cooling hole exceeds 2300, surpassing the critical value for turbulence onset, the flow is classified as turbulent. Owing to the small dimensions of the film cooling hole, the resulting turbulence may exhibit distinct flow phenomena, such as boundary layer transition and vortex–wall interactions. The large eddy simulation (LES) model is employed to resolve large-scale eddies directly while modeling small-scale eddies using a subgrid-scale approach. This method is particularly well-suited for capturing transient flow behavior and vortex structures in free shear and channel flows. Accordingly, this study utilizes the LES model to investigate the dynamic processes of vortex generation, shedding, and energy transfer within abrasive flow in micro-channels.

The core concept of the LES model lies in spatially filtering the Navier-Stokes equations to separate and directly compute the resolved scales, while modeling the subgrid-scale motions. The governing mathematical formulation is presented as follows: Firstly, the velocity field *u_i_* is filtered to obtain the filtered velocity field $\bar{u_{i}}$.

(18) $$\bar{u_{i}}\left(x,t\right)=\int G\left(x-x^{\prime }\right)u_{i}\left(x^{\prime },t\right)dx^{\prime }$$

where *G* is the filtering function, which is used to filter vortex that is smaller than the filter scale Δ, usually relating to the grid scale, $\Delta ={\left(\Delta x\Delta y\Delta z\right)}^{1/3}$. After filtering Equations (16) and (17), the Navier–Stokes equation and continuity equation for large eddy simulation are obtained.

(19) $$\frac{\partial {\bar{u}}_{i}}{\partial t}+\frac{\delta \left({\bar{u}}_{i}{\bar{u}}_{j}\right)}{\delta x_{j}}=-\frac{1}{\rho }\frac{\delta \bar{p}}{\delta x_{i}}+\nu \frac{\partial^{2}{\bar{u}}_{i}}{\partial {x_{i}}^{2}}-\frac{\delta \tau_{ij}}{\delta x_{i}}$$

(20) $$\frac{\delta {\bar{u}}_{i}}{\delta x_{i}}=0$$

where $\tau_{ij}$ is subgrid-scale stress tensor, representing the influence of unresolved small-scale vortices on the large-scale flow, $\tau_{ij}=\bar{u_{i}u_{j}}-{\bar{u}}_{i}{\bar{u}}_{j}$. The subgrid-scale stress tensor is usually closed using the eddy viscosity model, based on the Boussinesq hypothesis,

(21) $$\tau_{ij}-\frac{1}{3}\tau_{kk}\delta_{ij}=-2\nu_{t}{\bar{S}}_{ij}$$

where ${\bar{S}}_{ij}$ is the filtered strain rate tensor, and *ν_t_* is the subgrid-scale eddy viscosity. ${\bar{S}}_{ij}=\frac{1}{2}\left(\frac{\delta {\bar{u}}_{i}}{\delta x_{j}}+\frac{\delta {\bar{u}}_{j}}{\delta x_{i}}\right)$.

(22) $$\nu_{t}={\left(C_{s}\Delta \right)}^{2}|\bar{S}|$$

where *C_s_* is Smagorinsky constant, which is usually set to 0.1~0.2. $|\bar{S}|=\sqrt{2{\bar{S}}_{ij}{\bar{S}}_{ij}}$.

At the initial stage of abrasive flow machining, the tooling passages and film cooling hole are filled entirely with air. As a result, the abrasive flow is characterized as a gas–liquid two-phase flow. To model this behavior, the Volume of Fluid (VOF) method is adopted in the numerical simulation. A function *F*(*x*, *y, z*, *t*) is defined to track the gas–liquid interface, where *F* = 1 denotes a cell fully occupied by the liquid phase, and *F* = 0 indicates the gas phase.

(23) $$\frac{\partial F}{\partial t}+\frac{F}{V_{F}}A\cdot \nabla U=0$$

where *V_F_* is the fractional volume open to flow, and A is the fractional areas open to flow in x, y, and z directions.

When the abrasive flow rapidly enters the film cooling hole, it impinges on the inner wall, generating complex turbulent structures. Low-pressure regions readily form near vortices, potentially leading to cavitation. To capture this phenomenon, a cavitation potential model is incorporated into the numerical simulation, defined as follows:

(24) $$S=\int_{0}^{t}\max\left(0,\;P_{cav}-P_{i,j,k}\right)\;dt$$

where *P_cav_* and *P_i,j,k_* are the cavitation pressure and pressure in the computational cell, respectively. *t* denotes time. Regions exhibiting higher cavitation potential *S* are more prone to cavitation. The cavitation bubbles generated will be carried along with the abrasive flow, and their size and internal pressure satisfy the following relationship:

(25) $$P=P_{0}{\left(\frac{V_{B0}}{V_{B}}\right)}^{\varphi }$$

where *P*_0_ and *V_B_*_0_ are the initial pressure and volume of the bubble, respectively; *P* and *V_B_* are the real-time pressure and volume of the bubble, respectively; *φ* is the ratio of specific heats at constant pressure to that of constant volume for the void, *φ* = *C_P_*/*C_V_*.

By employing the numerical model established in this study, the locations of bubble formation and collapse can be simulated, thereby enabling the prediction of cavitation regions within the film cooling holes.

### 4.2. Model Solving and Validation

**Figure 5:**
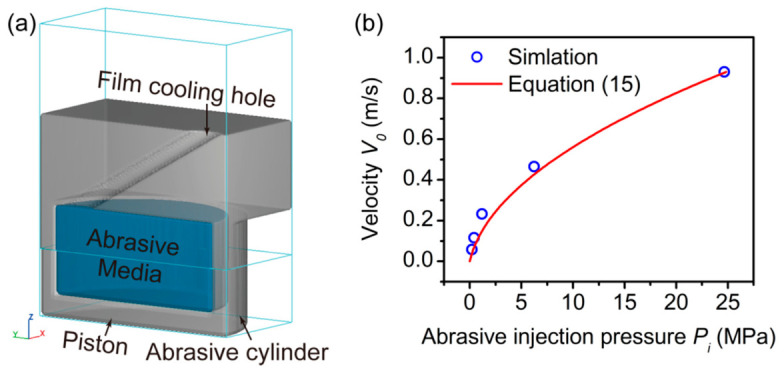
(**a**) Simulation model and (**b**) variation of polishing velocity *V*_0_ versus abrasive injection pressure *P_i_*.

Figure 5a shows the simulation model for abrasive flow machining of the film cooling hole. In the numerical model, the diameter of the abrasive cylinder piston *D_sim_* is 4.2 mm, while the diameter of the film cooling hole *d* is 0.4 mm, resulting in a cylinder-to-hole diameter ratio *k_sim_* = *D_sim_*/*d* = 10.5. An abrasive cylinder piston feed rate of 0.07 m/s was employed to ensure the consistency of the average flow speed of the abrasive media in the film cooling hole between the simulation model and the experimental conditions. In this work, the numerical models were solved by a commercial CFD software Flow-3D 10.1. To validate the accuracy of the numerical model, the independence of the average abrasive flow speed on abrasive injection pressure is characterized. As shown in Figure 5b, the simulation results closely match the theoretical predictions from Equation (15) when the pressure correction factor *δ* is set to 9. This confirms that the numerical model can reasonably capture the dynamic behavior of abrasive flow during the polishing of film cooling holes.

Here, in this work, the numerical model is validated through comparison with both the analytically measured pressure-velocity relationship and the observed locations and characteristics of polishing defects under representative processing conditions (demonstrated in subsequent corresponding sections). It should be noted that the present model is intended to resolve the evolution of the abrasive-flow field, including velocity distribution, wall shear stress, void formation, and cavitation potential, rather than to directly predict material removal or defect dimensions. Consequently, the validation focuses on the consistency between the predicted flow phenomena and the experimentally observed defect patterns. The agreement between the simulated high-shear and cavitation-prone regions and the corresponding defect locations provides support for the proposed defect-formation mechanisms.

## 5. Analysis of Polishing Defects

### 5.1. Features of Polishing Defects

In our experiments, the polishing performance of film cooling holes with different inclination angles (30°, 45°, and 60°) was investigated under two distinct polishing velocity conditions: a high velocity of *V_i_* = 0.033 m/s (*Re* = 1.4 × 10^5^~2.0 × 10^5^ at steady state) and a lower velocity of *V_i_* = 0.0043 m/s (*Re* = 4.1 × 10^3^~4.7 × 10^3^ at steady state). The Reynolds number inside the film cooling hole is defined as *Re* = *ρdV*_0_/*μ*, where *V*_0_ is the mean outlet velocity (Equation (3)), *d* is the hole diameter, and *μ* is an apparent viscosity determined from the measured pressure-flow characteristics using an equivalent pipe-flow formulation. The power-law fit (*μ* = 100.01*γ*^−0.4167^ mPa·s) shown in Figure 3 was established over the experimentally measured shear-rate range *γ* ≤ 100 s^−1^. Extrapolation of this relation to the wall shear rates encountered during polishing (*γ* ≈ 8*V*_0_/*d* ≈ 2 × 10^6^ s^−1^ for *V*_0_ = 102.8 m/s) falls outside the validity of the fitted constitutive model. Accordingly, the Reynolds numbers reported in this study are used as effective dimensionless indicators for comparing flow regimes rather than as conventional Reynolds numbers derived directly from the power-law viscosity.

**Figure 6:**
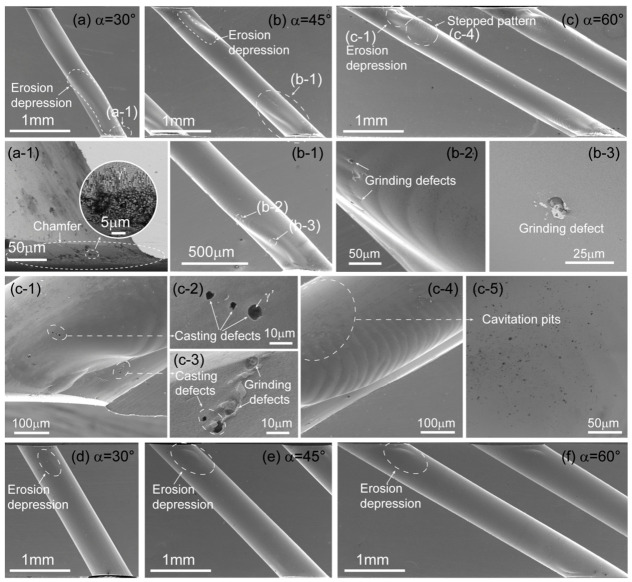
SEM images of the finished film cooling holes with different inclination angles under a high Reynolds number of 1.4 × 10^5^ to 2.0 × 10^5^ (**a**–**c**) and a lower Reynolds number of 4.1 × 10^3^~4.7 × 10^3^ (**d**–**f**): (**a**) overview in 30° regime; (**a-1**) chamfer microstructure; (**b**) overview with erosion depression in 45° regime; (**b-1**) magnified depression region; (**b-2**,**b-3**) magnified grinding defects; (**c**) overview with erosion depression and stepped pattern in 60° regime; (**c-1**) depression detail; (**c-2**,**c-3**) magnified casting and grinding defects; (**c-4**) stepped pattern/cavitation-pit region; (**c-5**) magnified cavitation pits. (**d**–**f**) Overviews for 30°, 45°, 60°, respectively, under low-velocity conditions, each with the erosion depression indicated.

The corresponding SEM images are shown in Figure 6, with Figure 6a–c representing high-velocity polishing regimes and Figure 6d–f corresponding to the low-velocity conditions. For each inclination angle-velocity combination, three of the eight nominally identical film cooling holes on each test specimen were sectioned and examined by SEM. The defect type and its spatial location (e.g., inlet-side erosion depression and trailing-edge stepped pattern) were consistent across all examined holes within the same test group. Variations in defect length were typically within 10% (Table 3), likely arising from minor differences in local edge sharpness and inlet geometry among the femtosecond-laser-drilled holes. The SEM images shown in Figure 6 are representative of the characteristic defect morphology observed under each processing condition.

The results reveal the formation of erosion depressions (Figure 6(b-1,c-1)) extending inward from the inlet of the film cooling holes. Under high-velocity conditions, the length of the erosion depression decreases with increasing inclination angle, while the depth increases, indicating a stronger abrasive cutting effect in this region. As a result, grinding-induced defects were observed within these regions, as shown in Figure 6(b-1–b-3). Additionally, casting defects became visible in the 60° inclined holes, as illustrated in Figure 6(c-1–c-3). In contrast, erosion depressions in the low-velocity regime (Figure 6d–f) were significantly shallower, and no grinding and casting defects were detected. The micro-topography further revealed the presence of stepped surface patterns extending from the trailing edge of the erosion depression in the 45° and 60° cases (Figure 6(b-2,c-4)). This phenomenon was absent in the 30° case (Figure 6a), where the hole evolved into an S-shaped channel, suggesting that the formation of stepped patterns becomes increasingly pronounced with greater inclination angles. Notably, no stepped features were observed under low-velocity conditions (Figure 6d–f).

Upon closer inspection, prominent plaque-like surface defects were observed on the chamfered inlet edges, particularly near the obtuse and acute angles, under high-velocity conditions. Microstructural analysis (Figure 6(a-1)) indicates exposure of the γ′ phase, likely due to preferential removal of the softer γ phase. Furthermore, cavitation pits were observed at the upper inlet of the 60° hole (Figure 6(c-4,c-5)), scattered along the outer region of the stepped patterns. These cavitation-induced defects were absent in all low-velocity polishing samples.

The SEM images shown in Figure 6 collectively demonstrate that distinct fluid dynamic behaviors emerge under different processing conditions, highlighting a strong correlation between flow characteristics and the resulting surface morphology of the film cooling holes. To supplement the SEM observations, the principal defect dimensions were measured directly from the micrographs and are summarized in Table 3. The measurements are consistent with the observed trends: under high-velocity polishing conditions, the erosion-depression length decreases whereas the defect depth increases with increasing hole inclination angle. In addition, stepped-pattern defects and cavitation pits were observed only at an inclination angle of 60° under high-velocity conditions. While, under low-velocity polishing conditions, the erosion-depression length and depth both exhibited a slight increasing trend with increasing hole inclination angle. Notably, stepped-pattern defects were absent across all tested angles under low-velocity conditions, suggesting that the formation of such morphological features is highly dependent on the flow velocity.

### 5.2. Formation Mechanisms of Polishing Defects

To investigate the formation mechanisms of polishing defects in film cooling holes, numerical simulations were conducted under conditions equivalent to those of the experiments. Both transient and steady-state fluid dynamics of abrasive flow within the holes were analyzed under high and low polishing velocities. Since the initial liquid level of the abrasive media in the experiment was lower than the clamping position of the test piece, the initial fluid elevation in the simulation was set to align with the inlet of the film cooling hole to reflect this physical condition. Moreover, due to the Froude number ($Fr=V_{0}/\sqrt{gd}$) being significantly greater than 1 in the polishing process, indicating that the gravity effect on fluid dynamics can be neglected, the flow behavior in the reverse stage was therefore assumed to mirror that of the forward stage in the initial half-cycle under the same initial condition. As a result, only the forward-direction flow, where abrasive media enters from one side and exits through the other, was simulated, despite the actual polishing cycle involving bidirectional flow.

**Figure 7:**
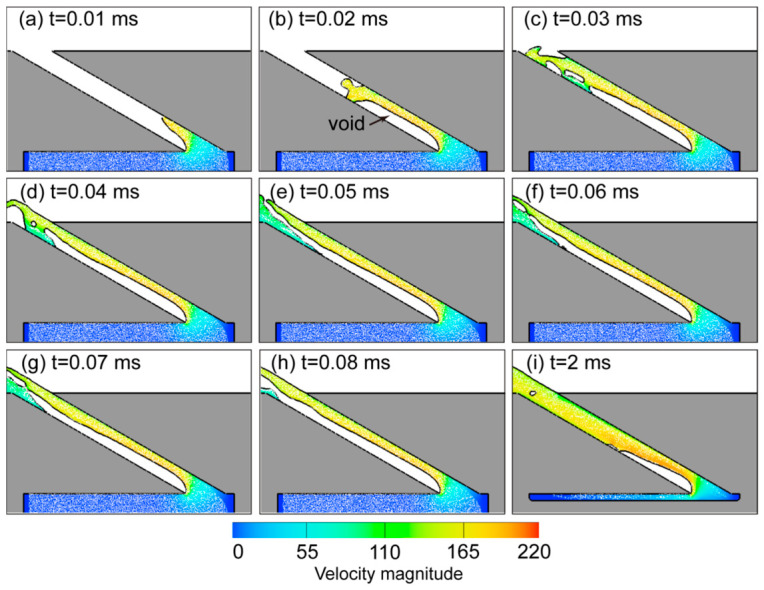
Velocity field in the film cooling hole with an inclination angle of 60° under a high polishing velocity condition (*Re* = 1.5 × 10^5^).

Figure 7 illustrates the simulated velocity field for a film cooling hole with a 60° inclination under a high Reynolds number condition (*Re* = 1.5 × 10^5^). In this figure, white dots represent SiC particles within the abrasive media. At 0.01 ms (Figure 7a), the abrasive flow enters the hole along the obtuse-edge side. As the flow progresses, the fluid front advances through the middle section while entrapping air near the inlet, forming a void region due to the flow constriction effect. Between 0.03 and 0.04 ms (Figure 7c,d), as the fluid front collides with the inner wall, bubbles begin to form. By 0.05 ms, the trapped air escapes, resulting in the formation of a narrow air cavity that connects to the external environment.

As the flow evolves, the air cavity gradually shrinks, and by 2 ms (Figure 7i), just before the abrasive media exits completely, a residual air pocket remains along the acute edge near the inlet. This unpolished wall in the void region is therefore only processed during the reverse flow stage of the polishing cycle. In contrast, the wall region subjected to flow constriction experiences strong shear forces due to the combined axial velocity and circumferential tangential velocity induced by the hole geometry. Consequently, an erosion depression gradually develops in this area, as shown in Figure 6(c-1), accompanied by localized acceleration–deceleration effects. As the abrasive media exits the depression zone, it transitions into a sheet-like flow, which contributes to the formation of stepped surface patterns, as illustrated in Figure 6(c-4). The stepped pattern is not an independent defect but a secondary, downstream consequence of the erosion depression. The depression forms first, at the location of peak combined axial–circumferential shear stress. Once it reaches sufficient depth, the abrasive stream exiting the depression separates from the wall and transitions into a thin, high-velocity sheet flow that abrades the adjacent, previously less-worn wall in a stepwise fashion, producing the stepped pattern immediately downstream of the depression’s trailing edge. Because this sheet-flow transition requires a sufficiently deep, energetic depression, stepped patterns appear only where the depression is well developed (45° and 60° under high-velocity conditions, Figure 6(b-2,c-4)) and are absent where the depression remains shallow (at 30°, where the flow instead evolves smoothly into an S-shaped channel, and under all low-velocity conditions).

**Figure 8:**
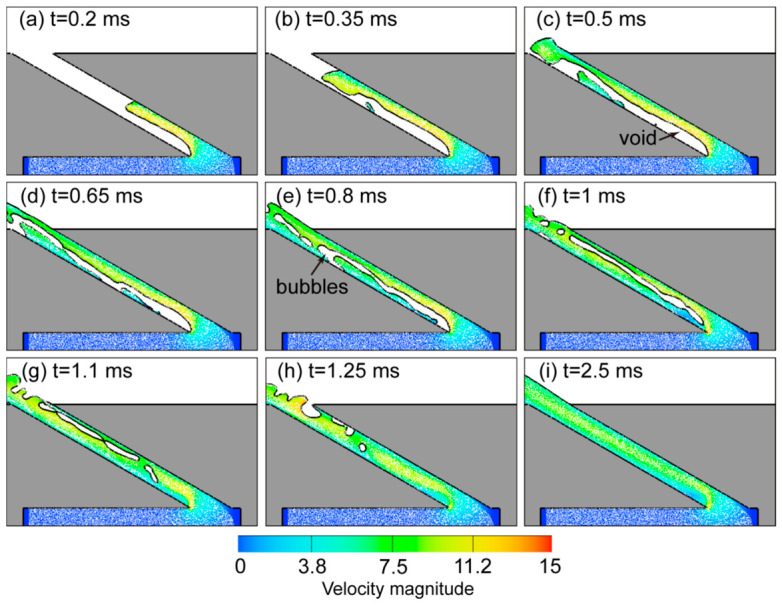
Velocity field in the film cooling hole with an inclination angle of 60° under a lower polishing velocity condition (*Re* = 3.0 × 10^3^).

In comparison, a significant lower polishing velocity regime was investigated. The Reynolds number Re was approximately 3 × 10^3^. As shown in Figure 8, during the period from 0.2 ms to 0.65 ms (Figure 8a–d), the fluid dynamic behavior is generally similar to that observed under high-velocity conditions. However, at 0.8 ms, the narrow air cavity fragments into several small bubbles and one elongated large bubble. In the subsequent period (0.8–1.25 ms, Figure 8f–h), the elongated bubble gradually rises with the abrasive flow and eventually disintegrates into smaller bubbles. At 1.1 ms and 1.25 ms (Figure 8g,h), these bubbles ascend to the surface and burst sequentially, resulting in the film cooling hole being completely filled with abrasive media. The steady-state flow field is shown in Figure 8i. Unlike the high-velocity regime illustrated in Figure 7, a low-speed vortex forms near the acute edge side of the inlet. This vortex exhibits reduced wall shear within its core, while the abrasive flow velocity increases along the outer edge of the vortex. As a result, an erosion depression also forms in this region, as depicted in Figure 6f. However, due to the overall lower flow velocity, the erosion depression is much less pronounced, and thus no sheet flow is produced at the trailing edge. This explains the absence of stepped surface patterns on the wall of the film cooling hole under low-velocity polishing conditions.

**Figure 9:**
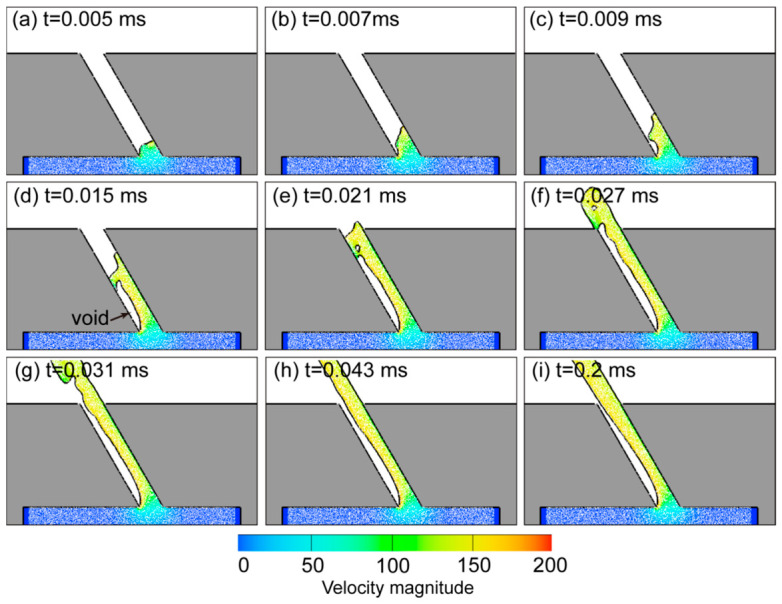
Velocity field in the film cooling hole with an inclination angle of 30° under a high polishing velocity condition (*Re* = 1.5 × 10^5^).

**Figure 10:**
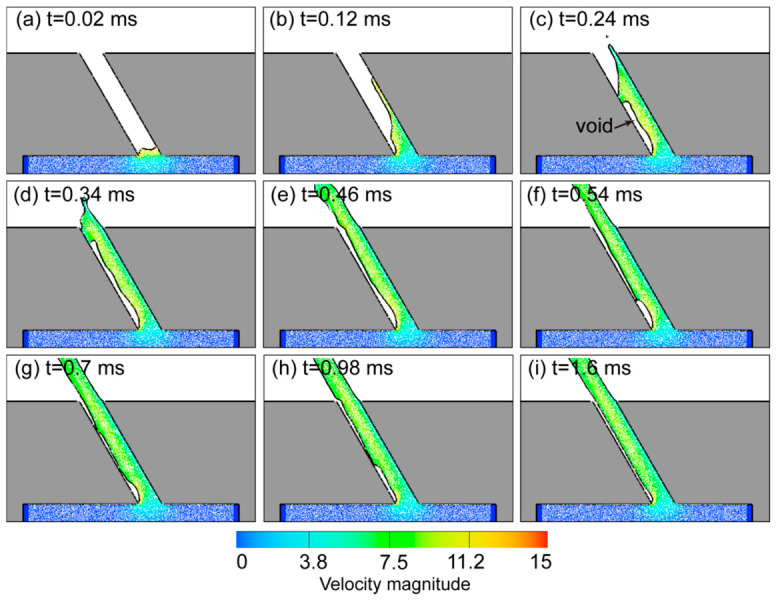
Velocity field in the film cooling hole with an inclination angle of 30° under a lower polishing velocity condition (*Re* = 3.0 × 10^3^).

Furthermore, given the noticeable differences in polishing results at varying inclination angles, the fluid dynamics during the polishing process of film cooling holes with a small inclination angle of 30° were investigated. Figure 9 and Figure 10 correspond to the high- and low-velocity polishing regimes, respectively. As shown in Figure 9a,b, the abrasive flow still enters the film cooling hole along the obtuse-edge side of the inlet. At 0.009 ms (Figure 9c), driven by the reaction force from the inclined wall, the abrasive flow spreads radially at the flow front. As a result, part of the air near the inlet becomes entrapped within the abrasive media, forming an air cavity on the acute-edge side. Between 0.009 ms and 0.027 ms (Figure 9c–f), the air cavity gradually stretches forward and eventually connects to the external environment at 0.031 ms (Figure 9g). The flow field then approaches a steady state, leaving behind an airfoil-shaped air pocket. Due to the smaller inclination angle, the constriction region in Figure 9 is significantly larger than that in Figure 7. Consequently, an elongated but shallow erosion depression is formed, as shown in Figure 6a, which suppresses the local acceleration-deceleration effects. Therefore, no stepped surface patterns are observed within the erosion region.

In contrast, under the low polishing velocity regime, as shown in Figure 10, the influence of the inclined wall on the abrasive flow is significantly reduced. The fluid front advances along the obtuse-edge side and nearly traverses the entire film cooling hole by 0.24 ms (Figure 10c). At this point, radial flow reaches the acute edge, forming an air cavity similar to those observed in previous cases. Subsequently, the cavity elongates as the abrasive continues to flow. By 0.46 ms (Figure 10e), the cavity connects with the external environment. From 0.54 ms (Figure 10f) to 0.70 ms (Figure 10g), the void region gradually contracts. However, at 0.98 ms (Figure 10h), a residual air pocket elongates again, forming a narrow air channel that extends through the film cooling hole wall, as shown in Figure 10i. As a result, the circumferential tangential shear force exerted on the wall is minimal. Consequently, no significant erosion depression is observed on the wall of the film cooling hole, as illustrated in Figure 6d.

**Figure 11:**
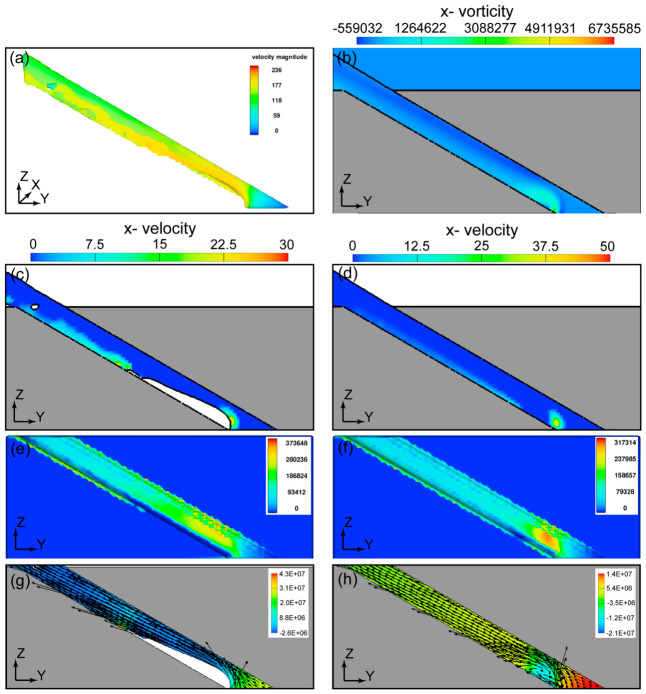
3D velocity magnitude (**a**), velocity contour in x-direction (**c**), shear stress on the wall (**e**) and instantaneous pressure field (Pa) and velocity vectors (**g**) during the steady-state polishing process for the standard case analogous to Figure 7 (*t* = 2 ms). In contrast, (**b**,**d**,**f**,**h**) respectively depict the vorticity intensity, velocity contour in x-direction and wall shear stress for the modified (elevated-initial-level) case analogous to Figure 7.

To summarize, in abrasive flow finishing of film cooling holes, increases in both inclination angle and polishing velocity introduce multiple sources of uncertainty. As the abrasive media enters the film cooling hole, it accelerates due to the reduction in channel cross-section and the constriction effect. The high-velocity abrasive flow primarily impinges on the inner wall of the hole, while simultaneously forming extensive void regions within the flow field, as illustrated in Figure 11a. Influenced by the curved surface of the film cooling hole, part of the incident flow disperses circumferentially, as shown in Figure 11c, generating substantial shear forces along the wall in the x-direction, as depicted in Figure 11e.

Notably, the flow dynamics are strongly affected by the initial fluid level. To explain this, a modified polishing process analogous to that in Figure 7 is examined. The experimental conditions remain identical to Figure 7 configuration, except for a raised initial fluid level that exceeds the film-cooling-hole inlet. We found that the resulting flow behavior differs significantly. The flow field rapidly stabilizes, and no void regions are observed, as shown in Figure 11b. Instead of a void, a vortex forms in the same location previously occupied by the cavity (Figure 11c). As illustrated in Figure 11b,d, the vorticity intensity in the x-direction is substantial, leading to an increase in the x-direction flow velocity and inducing strong local shear stress in the vortex region. A comparison between the high-shear regions in Figure 11e,f shows a direct correlation with the erosion depression geometries observed at the lower and upper ends of the film cooling hole, respectively, as shown in Figure 6c. The shear force contours closely correspond to the geometry of the asymmetrical polishing-induced defects observed at opposite ends of the film cooling hole. In fact, these two distinct flow behaviors represent two different initial experimental conditions in a polishing cycle. In the forward stage of the polishing cycle, the abrasive level is initially below the fixture-clamped film cooling hole. In contrast, during the reverse stage, the hole is fully filled with abrasive media, owing to gravitational effects hindering complete evacuation of residual abrasive media from the previous cycle. The corresponding pressure fields for the standard and modified cases are presented in Figure 11g,h. The lower-end (standard-case) depression produces a longer stepped pattern, whereas the upper-end (modified-case) depression produces a shorter one. As indicated by the velocity vectors in Figure 11g,h, this difference arises from the way the abrasive stream separates from the wall after leaving the erosion depression. In the standard case (Figure 11g), the flow remains nearly parallel to the wall and deviates only slightly near the downstream end of the persistent void, allowing the sheet flow to remain attached over a relatively long downstream distance and thereby generating an extended stepped pattern. In the modified case (Figure 11h), the flow turns abruptly at the acute-edge outlet corner, where a low-pressure vortex core forms adjacent to a high-pressure stagnation region. The sheet flow therefore detaches from the wall more rapidly, limiting the downstream abrasion distance and producing a shorter, more localized stepped pattern. The localized low-pressure region associated with the vortex also favors cavitation inception, explaining why cavitation pits are observed together with the upper-end stepped pattern (Figure 6(c-4,c-5)) but are absent from the lower-end stepped pattern. In this section, the agreement between the simulated fluid dynamics and the observed polishing defects further confirms the validity of the numerical model.

In addition, two distinct gas-phase features are identified in the simulations and are distinguished here to clarify the terminology used throughout this study. The first is a persistent air cavity formed during the transient filling stage as a result of incomplete wetting. This cavity is identified by the VOF volume fraction field (F = 0) (Figure 7, Figure 8, Figure 9 and Figure 10) and persists or is displaced as the abrasive front advances, without undergoing collapse. The second feature is cavitation, as defined by Equations (24) and (25), which occurs when the local pressure within a vortex core falls below the cavitation pressure, leading to the nucleation of microbubbles. These vapor microbubbles are subsequently transported into regions of higher pressure near the vortex periphery, where they collapse. The cavitation pits observed by SEM (Figure 6(c-4,c-5)) are therefore attributed to microbubble-collapse events rather than to the persistent air cavity. Direct observation of individual bubble nucleation and collapse within the sub-millimeter film cooling hole was not feasible using the present experimental setup. Consequently, this interpretation is based on the spatial agreement between the predicted regions of high cavitation potential and the experimentally observed pit locations, providing mechanistic support rather than direct experimental verification.

Experimental results demonstrate that material removal is most significant in regions of strong shear, leading to the formation of erosion depressions and, in some cases, grinding-related defects. As both inclination angle and polishing velocity increase, shear forces intensify, resulting in deeper erosion depressions and the exposure of internal casting defects. Additionally, the low-pressure core of the vortex facilitates cavitation, producing bubbles within the abrasive flow. When these bubbles are transported into high-pressure regions near the vortex periphery, their collapse gives rise to cavitation pits, as shown in Figure 6(c-5). This behavior is consistent with observations reported for Venturi-type cavitation reactors, where cavitation development is governed by the interaction between vortex structures and local pressure fluctuations [30]. In the present film-cooling-hole configuration, the acute-edge vortex serves a similar function to the throat vortex in a Venturi device. The vortex core maintains a region of reduced pressure that promotes bubble inception, whereas the subsequent pressure recovery in the surrounding flow field induces bubble collapse. Unlike Venturi geometries, where cavitation collapse is largely associated with the throat-diffuser transition, bubble collapse in the present case occurs near the periphery of the vortex structure, leading to localized cavitation-pit formation. This vortex–pressure-coupling perspective reinforces the mechanistic link between the simulated flow field and the cavitation-pit defects observed experimentally (Figure 6(c-5)).

Although this study distinguishes cavitation regimes qualitatively through the two characteristic flow behaviors described above (void-dominated versus vortex-dominated, depending on initial fluid level). Data-driven modal decomposition methods such as Dynamic Mode Decomposition (DMD), recently used to classify cavitating flow regimes from time-resolved flow-field data [31], offer a way to formalize this distinction quantitatively using the transient VOF/LES output already generated by the present model. These methods represent as a promising direction for future analysis of the cavitation dynamics reported here.

### 5.3. Impacts of Processing Parameters on Polishing Defects

As demonstrated in the preceding analysis, the polishing quality of film cooling holes is closely related to the fluid dynamics, which are strongly influenced by the initial conditions, hole geometry, and physical properties of the abrasive media. To elucidate the effects of key processing parameters on polishing performance, the relationships between fluid dynamics and factors such as film cooling hole thickness, inclination angle, abrasive particle size, abrasive viscosity, and polishing velocity were systematically investigated. It should be noted that the parameter analyses presented in this section are intended to reveal the influence of processing parameters on the underlying flow dynamics and defect-formation tendency. They should not be interpreted as direct predictions of material removal or final defect dimensions, which would require a coupled material-removal model and dedicated experimental validation.

#### 5.3.1. Effects of Polishing Velocity

**Figure 12:**
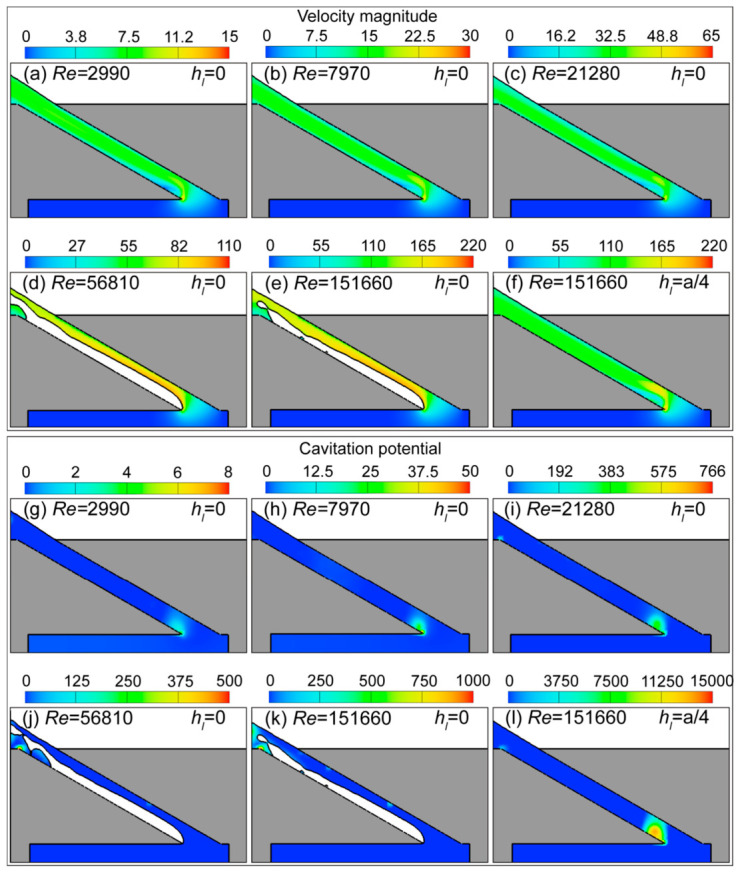
Velocity magnitude (**a**–**f**) and cavitation potential (**g**–**l**) of the abrasive flow in the film cooling hole at steady state.

In abrasive polishing, the cutting force is generated by the shear force exerted by the abrasive flow on the wall of the film cooling hole. This shear force is closely related to the shear rate of fluid. Therefore, the abrasive flow velocity (normalized as Reynolds number *Re*) is a critical factor that affects the polishing quality. In our simulations, we investigated the velocity magnitude (Figure 12a–f) and cavitation potential (Figure 12g–l) under various *Re* conditions. As shown in Figure 12a–e, the flow field becomes increasingly chaotic with rising *Re*. When *Re* exceeds 56,810, the abrasive flow is unable to fully wet the entire wall surface due to high inertial forces, resulting in the formation of a narrow air cavity clinging to the acute edge of the hole. As noted in the previous section, when the initial fluid level of the abrasive media exceeds the inlet height of the film cooling hole, the resulting velocity field (Figure 12f) differs significantly from that in Figure 12e, but closely resembles the patterns observed in Figure 12a–c, despite having the same *Re* value. This indicates that initial fluid level plays a critical role in flow behavior, beyond what can be explained by Reynolds number alone.

The cavitation potential of the abrasive flow was also investigated. This parameter represents the density of microbubbles smaller than the computational mesh size, where a higher cavitation potential signifies a more severe cavitation effect. As shown in Figure 12g–l, cavitation potential is most prominent within the vortex regions. Additionally, when *Re* exceeds 21,280 (corresponding to an outlet velocity *V*_0_ ≈ 30 m/s and an injection pressure *P_i_* ~ 3.5 MPa), notable cavitation potential appears at the edge-rounded surface near the obtuse-edge side of the outlet. This localized effect contributes to intensified abrasion in that region, which is consistent with the experimental observations shown in Figure 6(a-1).

**Figure 13:**
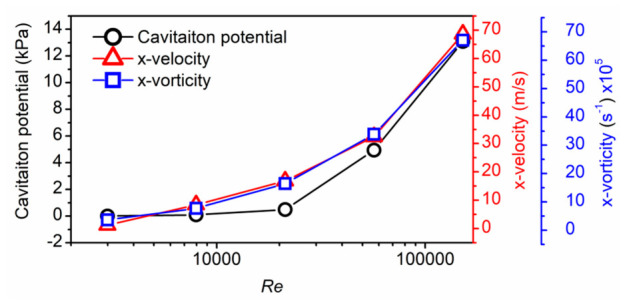
Maximum cavitation potential, x-velocity and x-vorticity of the abrasive flow in vortex regions under different Reynolds number conditions.

Furthermore, the dependence of the maximum cavitation potential, x-velocity and x-vorticity of the abrasive flow at the vortex areas on *Re* number were characterized, as shown in Figure 13. Due to the discontinuous nature of the flow field at very high Reynolds numbers (Figure 12d,e), the corresponding values were evaluated using an alternative flow condition, as represented by the case in Figure 12f. Figure 13 demonstrates that the cavitation potential, x-velocity and x-vorticity intensity all increase with increasing Reynolds number. Notably, when the *Re* exceeds 21,280, these indicators exhibit a marked rise. This abrupt increase clearly undermines the uniformity of material removal during AFM finishing of film cooling holes, leading to undesirable variations in surface quality. This regime-transition behavior, where cavitation potential rises sharply once a critical Reynolds number is exceeded rather than increasing gradually is consistent with the threshold-dependent cavitation intensity changes reported by Ge et al. [32] for Venturi-type cavitation reactors under varying operating conditions, suggesting that this transition-dominated character is a general feature of vortex-driven hydrodynamic cavitation.

#### 5.3.2. Effects of Film Cooling Hole Structural Features

In practical applications, the inclination angle and aspect ratio of film cooling holes on turbine blades vary significantly due to the curved surface geometry and the complexity of internal flow channels. Therefore, it is of great significance to investigate the fluid dynamics of abrasive flow within film cooling holes featuring different geometrical configurations. In practical turbine blades, the curved surface geometry and the constrained internal flow path jointly determine the local hole inclination angle and the effective aspect ratio. Therefore, these two parameters are selected as representative geometric descriptors to quantify the influence of hole structure on abrasive-flow polishing quality.

**Figure 14:**
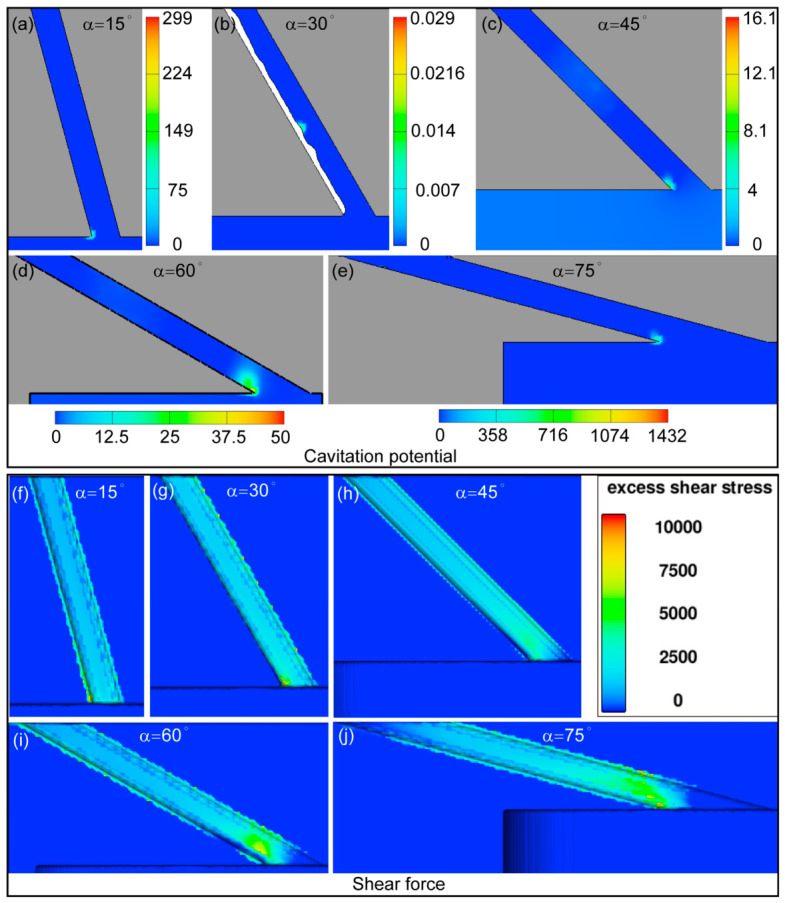
(**a**–**e**) Cavitation potential contours of the abrasive flow and (**f**–**j**) contours of the shear force on the wall of the film cooling hole with different inclination angles.

**Figure 15:**
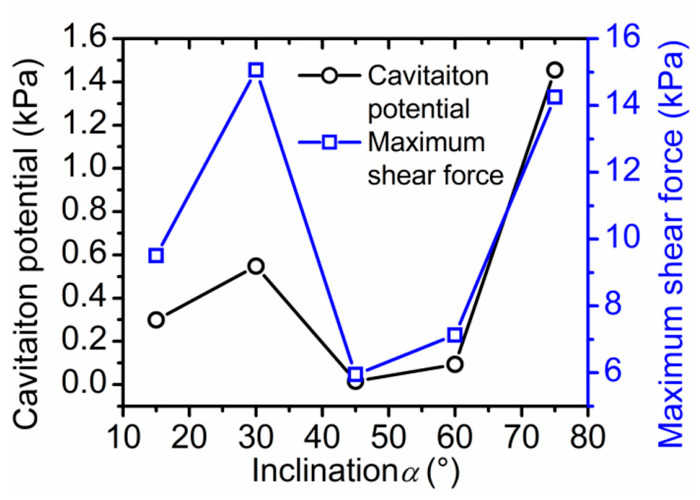
Cavitation potential and maximum shear force on the wall of the film cooling hole with different inclination angles.

Figure 14a–e shows the steady-state cavitation potential contours of the abrasive flow at a Reynolds number of 7910. When the inclination angle is small, the cavitation region remains relatively localized near the acute edge. As the inclination angle increases, the cavitation zone expands and gradually extends deeper into the internal flow channel. However, when the inclination angle increases further from 60° to 75°, the cavitation area diminishes significantly. An exception is observed in the film cooling hole with a 30° inclination angle, where a narrow air cavity forms within the abrasive flow. This phenomenon persists even under lower Reynolds number conditions, as shown in Figure 10. Additionally, Figure 14f–j illustrate the wall shear force distributions at different inclination angles. At lower inclination angles, strong shear forces are concentrated near the acute edge. As the inclination angle increases, the location of vortex formation shifts, resulting in intensified shear forces along the inner wall of the film cooling hole. As shown in Figure 15, the cavitation potential and maximum wall shear force (which contribute to excessive local material removal) initially decrease and then increase with the inclination angle. A local peak is observed at the 30° inclination condition, where the cavitation potential value is taken from the corresponding case with a higher initial fluid level exceeding the inlet. This suggests that both excessive and insufficient inclination angles of the film cooling hole are detrimental to achieving good polishing quality. An optimal angle between the film cooling hole and the surface normal is typically in the range of 45–60°.

**Figure 16:**
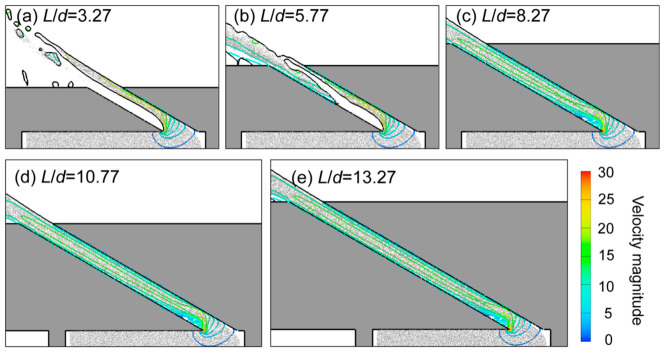
Steady-state velocity fields in the film cooling hole with different aspect ratios.

Figure 16 illustrates the steady-state velocity fields within film cooling holes of varying aspect ratios at a Reynolds number of 7910. In the experiment, the aspect ratio was adjusted by varying the thickness of the test piece. As shown in Figure 16a,b, when the aspect ratio is relatively small, the abrasive flow fails to fully stabilize, and the air initially present within the film cooling hole is difficult to remove. A smaller aspect ratio corresponds to a larger air cavity. However, when the aspect ratio exceeds 8.27, no bubbles are observed in the abrasive flow under steady-state conditions. Based on the velocity contour lines shown in Figure 16c–e, the influence of aspect ratio on the overall flow behavior appears to be relatively limited.

**Figure 17:**
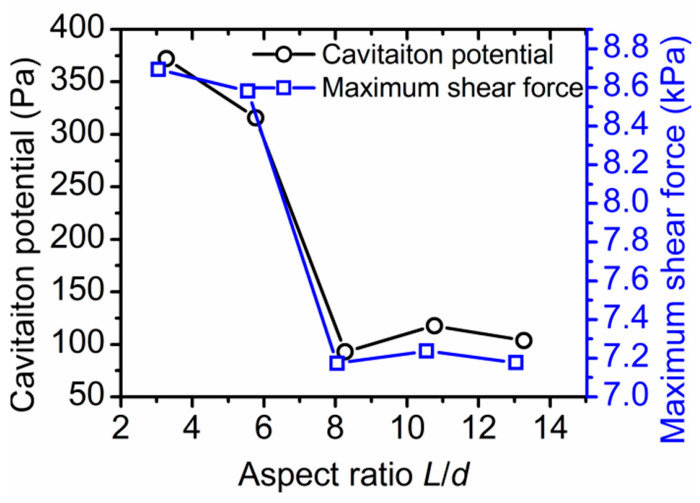
Cavitation potential and maximum shear force on the wall of the film cooling hole with different aspect ratios.

Figure 17 quantitatively illustrates the effect of aspect ratio on cavitation potential and wall shear force. Similar to the previous cases, the cavitation potential values for aspect ratios of *L*/*d* = 3.27 and *L*/*d* = 5.77 were obtained from simulations in which the initial liquid level exceeded the inlet of the film cooling hole. As shown in Figure 17, both cavitation potential and maximum shear force decrease with increasing aspect ratio. When the aspect ratio exceeds 8.27, these values drop sharply. However, as the aspect ratio increases further from 8.27 to 13.27, both cavitation potential and shear force remain nearly constant. This suggests improved polishing uniformity within this range of aspect ratios.

#### 5.3.3. Effects of Abrasive Particle Size

In addition to polishing velocity and film cooling hole geometry, the properties of abrasive particles represent another critical factor influencing polishing quality. For example, the material type and concentration of abrasive particles have been shown to significantly affect polishing performance. SiC and diamond abrasives exhibit distinct behaviors in brittle material machining due to differences in hardness, morphology, and chemical interactions. SiC, with a Vickers hardness of 28–34 GPa, features a polyangular morphology that promotes stress concentration at contact points, facilitating brittle fracture and efficient removal of surface protrusions [33]. However, its sharp edges contribute to variability in scratch depth, resulting in higher post-polishing surface roughness compared to spherical abrasives [34]. In contrast, diamond abrasives (Vickers hardness 70–100 GPa) offer superior initial material removal rates but are susceptible to chemical degradation when used with nickel-based alloys. This is attributed to the formation of the Ni_3_C phase [35], which accelerates abrasive dulling. Abrasive concentration also has a nonlinear effect on polishing efficiency: low concentrations (<35 wt.%) lead to discrete cutting and deep scratches due to insufficient particle density, while medium concentrations (35–55 wt.%) optimize kinetic energy transfer for uniform material removal. At high concentrations (>55 wt.%), increased particle collisions and agglomeration reduce slurry mobility and lead to the formation of micro-defects. These observations underscore the importance of balancing abrasive properties and operational parameters in precision machining applications [34].

**Figure 18:**
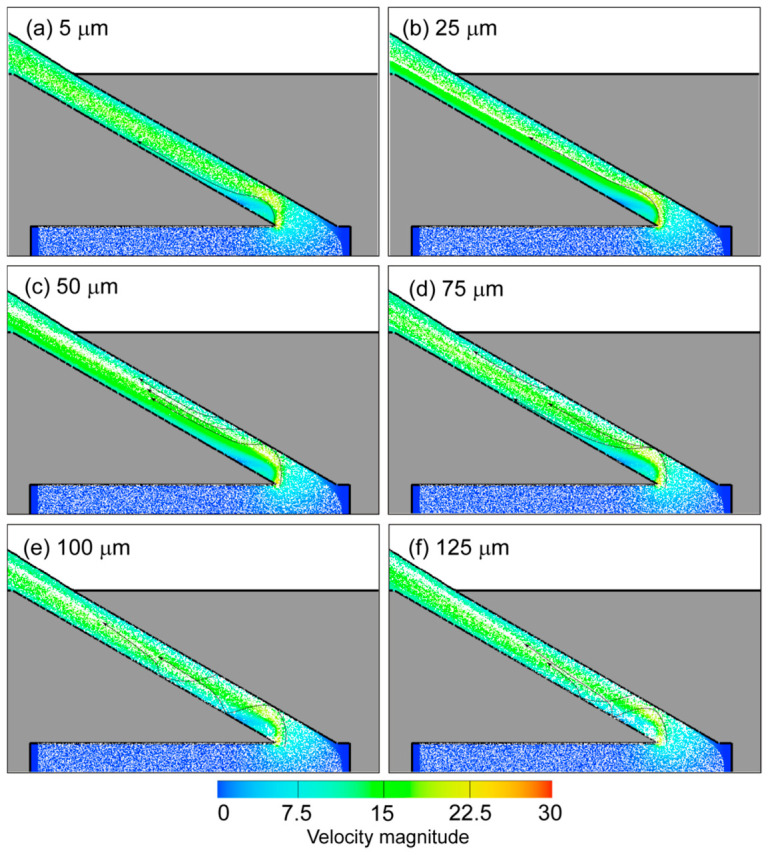
Particle distribution in the flow field with different abrasive size. The arrows in the figure indicate the direction of particle transport.

In this study, we investigated the effects of abrasive particle size (ranging from 5 μm to 125 μm) on particle distribution within the abrasive flow at a Reynolds number of 7910. As shown in Figure 18, particle distribution varied significantly with size. For smaller particles (Figure 18a), the distribution was nearly homogeneous throughout the flow field, except within the vortex region. At medium particle sizes (5–75 μm; Figure 18b,d), a distinct stratification was observed, with particles concentrated in the upper layers of the flow due to increased inertial effects. For larger particles (>75 μm; Figure 18d–f), particle inertia further increased, intensifying collisions with the film-cooling-hole wall. As particle size increased, rebound motion became more pronounced. After multiple impacts, a significant number of particles migrated toward a flow path near the axis. Moreover, large abrasive particles tended to accumulate in vortex regions. Based on these observations, it can be concluded that smaller abrasive particles are more favorable for achieving improved surface roughness and enhanced polishing uniformity on the inner walls of film cooling holes.

#### 5.3.4. Effects of Abrasive Viscosity

**Figure 19:**
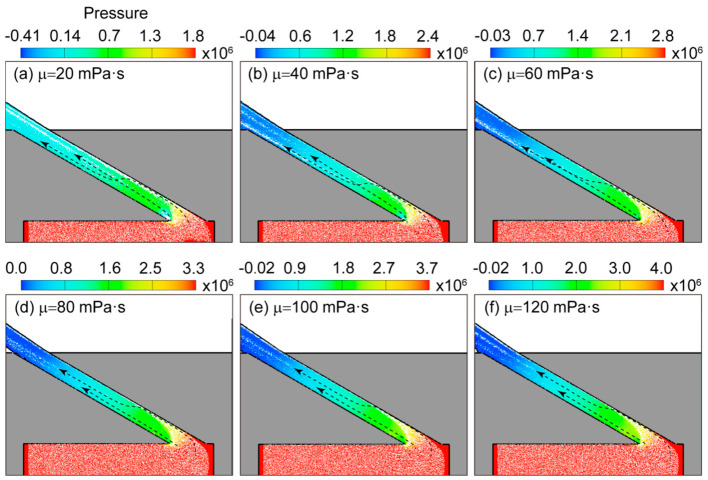
Particle distribution in the flow field at different abrasive viscosities. The arrows in the figure indicate the direction of particle transport.

Finally, the influence of abrasive viscosity on the flow field was investigated. Here, the abrasive viscosity was treated as a constant parameter, ranging from 20 to 120 mPa·s. Therefore, the Reynolds number was below 2300, and the abrasive flow was classified as laminar. The particle size was fixed at 100 μm for all viscosity conditions. As shown in Figure 19, to maintain a consistent polishing velocity across varying viscosities, the system pressure should be increased proportionally to the viscosity. As illustrated in Figure 19a, significant particle aggregation occurs within the vortex region at low viscosity, which is progressively suppressed as the viscosity increases from 40 mPa·s to 120 mPa·s (Figure 19b–f). Three distinct abrasive particle motion paths were identified within the flow field. The first path corresponds to particles flowing against the film-cooling-hole wall. The second path arises from particles that rebound off the wall surface. Notably, higher viscosity leads to shorter rebound distances. The third path consists of particles accumulating near the acute edge, a behavior that is largely unaffected by viscosity.

**Figure 20:**
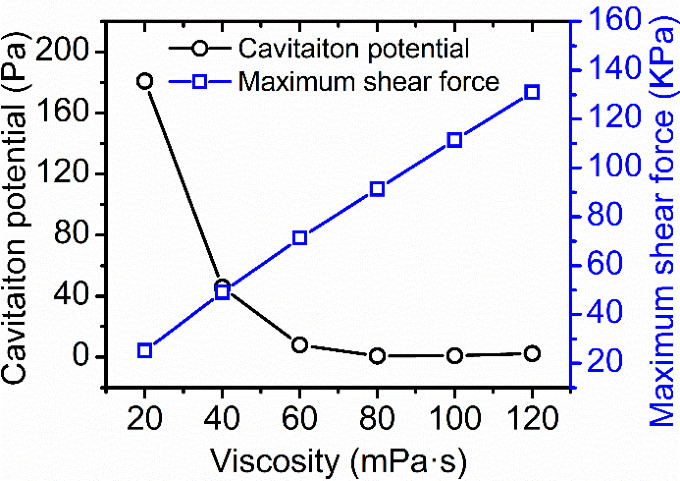
Variations of cavitation potential and maximum shear force versus abrasive media viscosity.

Moreover, the effects of abrasive media viscosity on cavitation potential and wall shear force were analyzed, as shown in Figure 20. The results indicate that cavitation potential decreases with increasing viscosity, while the maximum wall shear force increases. Therefore, the viscosity of the abrasive media must be carefully optimized to balance cavitation suppression and the risk of localized over-polishing. A viscosity of approximately 60 mPa·s appears to offer a reasonable compromise for effective abrasive media formulation. This value reflects a compromise among particle transport, cavitation suppression, and wall shear force, as illustrated in Figure 19 and Figure 20. The cavitation potential decreases rapidly as the viscosity increases to approximately 40 mPa·s and remains close to zero at higher viscosities, whereas the wall shear force increases continuously and rises more rapidly beyond this point. Consequently, the viscosity range of 40–60 mPa·s provides favorable conditions by effectively suppressing cavitation while avoiding a substantial increase in wall shear force. Within this range, a viscosity close to 60 mPa·s is preferred because it also reduces the likelihood of particle agglomeration in the vortex region (Figure 19). The optimum viscosity range is expected to vary with hole geometry and particle size because these factors influence the flow resistance, vortex structure, and particle inertia. Therefore, the viscosity values reported here should not be regarded as universally applicable. Instead, the design principle established in this study, which balances particle transport, cavitation suppression, and wall shear force using the combined analysis presented in Figure 19 and Figure 20, can be extended to other geometries and processing conditions.

#### 5.3.5. Integrated Discussion of Parameter Interactions

The favorable parameter ranges identified in Section 5.3.1, Section 5.3.2, Section 5.3.3 and Section 5.3.4 are interdependent rather than orthogonal. Although each parameter was examined separately, the underlying flow physics indicates several qualitative interactions that are relevant to process design.

An increase in aspect ratio increases hydraulic resistance within the film cooling hole and therefore places greater demands on the driving pressure required to maintain sufficient flow momentum. For this reason, operating toward the upper part of the recommended Reynolds-number range may be advantageous for longer holes. However, Section 5.3.1 and Section 5.3.3 show that excessively high Reynolds numbers also intensify wall shear stress fluctuations and increase the cavitation tendency, indicating a trade-off between maintaining adequate flow penetration and suppressing flow-induced defects.

Particle size and medium viscosity exhibit a similar coupling. Smaller abrasive particles improve transport through narrow passages and reduce the likelihood of localized blockage, whereas an appropriate medium viscosity is still required to maintain particle suspension and effective momentum transfer. Excessively low viscosity weakens particle-carrying capability, while excessively high viscosity increases flow resistance and limits the accessibility of the abrasive medium to the polishing region. Consequently, the favorable viscosity identified in the present study should be interpreted as the optimum within the investigated operating conditions rather than as an independent universal value. It should also be noted that reducing particle size lowers the kinetic energy per impact event, which implies a trade-off between improved surface uniformity and overall processing time unless compensated for a higher cycle count or a modestly increased pressure.

A larger inclination angle increases the effective path length and alters the local curvature distribution, which can intensify particle–wall impingement near the inlet unless the media viscosity is sufficient to dampen velocity fluctuations. This suggests that the recommended 45–60° range may interact with viscosity and particle-size selection: a more viscous medium may be needed to stabilize the flow in highly inclined holes, whereas a lower viscosity might suffice for shallower angles.

The present CFD analysis was designed to establish the qualitative influence of individual process parameters on the flow field and defect formation. Determining the globally optimal combination of Reynolds number, inclination angle, aspect ratio, particle size, and medium viscosity requires simultaneous multi-factor optimization together with quantitative prediction of material removal and experimental validation. This remains an important direction for future work.

## 6. Conclusions

This study presents a comprehensive investigation into the formation mechanisms of finishing defects during abrasive flow machining of turbine blade film cooling holes. Through integrated experimental and numerical analyses, three primary defect types (erosion depressions, stepped patterns, and cavitation pits) were identified and attributed to specific fluid dynamic behaviors within the curved micro-channels. Under the experimental and numerical conditions investigated in this study, a critical processing parameter window is suggested as follows, rather than as a universal process limit:

(1) Cavitation potential, non-uniform shear forces on the film-cooling-hole surface, and abrasive distribution are three critical factors contributing to the formation of cavitation pits, erosion depressions, and stepped pattern defects.
(2) To prevent excessive cavitation potential and local shear forces from causing polishing erosion depressions and cavitation pits, the Reynolds number of the abrasive flow within the film cooling hole should not exceed 2 × 10^4^.
(3) Both excessive and insufficient inclination angles of the film cooling hole are detrimental to achieving good polishing quality. An optimal angle between the film cooling hole and the surface normal is typically in the range of 45–60°.
(4) The aspect ratio of the film cooling hole should not be too low, as it may lead to excessive cavitation potential and localized shear forces. The minimum aspect ratio should be no less than 8.
(5) Based on numerical simulation, excessive particle diameter is predicted to hinder the uniform distribution of abrasive particles in the abrasive flow. Medium-sized abrasive particles (5–75 μm) tend to cause a stratification phenomenon, while larger particles (>75 μm) are predicted to accumulate on one side of the vortex region. On the other side, collisions between abrasive particles and the film-cooling-hole wall become more intense, which is unfavorable for improving the surface roughness of the hole wall.
(6) Numerical simulation indicates that higher abrasive viscosity effectively suppresses turbulent behavior and particle agglomeration, at the cost of requiring higher hydraulic pressure. A viscosity of approximately 60 mPa·s is predicted to offer a reasonable compromise for abrasive media formulation.

These results culminate in a set of process design guidelines aimed at minimizing surface damage while maximizing material removal efficiency. The research fills a critical knowledge gap in the understanding of surface defect formation in complex AFM applications. The insights gained provide a theoretical and technical foundation for precision finishing of micro-scale film cooling structures in nickel-based superalloy turbine blades.

## Figures and Tables

**Table 1:** Geometric parameters of film-cooling-hole specimens.

Parameters	Values
Number of holes per specimen	8
Hole diameter *d* (mm)	~0.4
Inclination angle (to surface normal) *α* (°)	30°, 45°, or 60° (per test group)
Specimen thickness *a* (mm)	~2
Effective hole length *L* (mm)	~2.1, 2.4, 3.3 corresponding to 30°, 45°, or 60°, respectively
Aspect ratio *L*/*d*	~5.2, 6.1, 8.3 corresponding to 30°, 45°, or 60°, respectively
Inlet/outlet edge condition	Sharp (as-drilled), acute and obtuse edges
Drilling method	Femtosecond laser drilling
Material of film-cooling-hole specimens	Nickel-based single-crystal superalloy

**Table 2:** Physical parameters of the abrasive media.

Physical Parameters	Values
Density *ρ* (kg/m^3^)	1544.2
Viscosity *μ* (mPa·s)	Figure 3
Abrasive media material	Water
Abrasive particle material	SiC
Abrasive particle size (μm)	0.4–5
Abrasive particle mass fraction (%)	50
Power-law index *n*	0.583
Consistency index *K* (Pa·s^n^)	0.1
Reference apparent viscosity, *μ* (*γ*~0.1 s^−1^) (mPa·s)	300

**Table 3:** Geometric characterization of defects in AFM-polished specimens.

Inclination Angle	Velocity	Depression Length (μm)	Depression Depth	Stepped-Pattern Extent (μm)
30°	High	1299 ± 23	Shallow	Not observed
45°	High	556 ± 33	Moderate	344 ± 2
60°	High	319 ± 7	Deep	659 ± 11
30°	Low	318 ± 29	Negligible	Not observed
45°	Low	343 ± 38	Minor	Not observed
60°	Low	361 ± 20	Limited	Not observed

## Data Availability

The original contributions presented in this study are included in the article. Further inquiries can be directed to the corresponding authors.
